# Freshness Evaluation of Three Kinds of Meats Based on the Electronic Nose

**DOI:** 10.3390/s19030605

**Published:** 2019-01-31

**Authors:** Jun Chen, Juanhong Gu, Rong Zhang, Yuezhong Mao, Shiyi Tian

**Affiliations:** 1 Inspection and Quarantine Integrated Technology Center, Suzhou Entry-Exit Inspection and Quarantine Bureau, Suzhou 215104, Jiangsu, China; chenjuneau@163.com (J.C.); gujh2013@126.com (J.G.); doit2003@126.com (R.Z.); 2 School of Food Science and Biotechnology, Zhejiang GongShang University, Hangzhou 310018, Zhejiang, China; myz-001@163.com

**Keywords:** freshness evaluation, meat, electronic nose

## Abstract

The aim of this study was to use an electronic nose set up in our lab to detect and predict the freshness of pork, beef and mutton. Three kinds of freshness, including fresh, sub-fresh and putrid, was established by human sensory evaluation and was used as a reference for the electronic nose’s discriminant factor analysis. The principal component analysis results showed the electronic nose could distinguish well pork, beef and mutton samples with different storage times. In the PCA figures, three kinds of meats samples all presented an approximate parabola trend during 7 days’ storage time. The discriminant factor analysis showed electronic nose could distinguish and judge well the freshness of samples (accuracy was 89.5%, 84.2% and 94.7% for pork, beef and mutton, respectively). Therefore, the electronic nose is promising for meat fresh detection application.

## 1. Introduction

Meats like pork, beef and mutton, which have high nutritional value and good taste, are some of the most important kinds of food in humans’ daily life [1,2]. However, due to its high nutrient substance concentration, meat is highly susceptible to spoilage and contamination. The freshness of meat degrades because microbial spoilage and biochemical reactions occur during storage. The carbohydrate, protein and fat will be decomposed into acetaldehyde, hydrogen sulfide and ammonia via the actions of bacteria and enzymes [3,4,5]. Therefore, meat with different freshness status will generate different kinds of gases.

In the traditional detection area, human sensory evaluation, chemical substances detection and microbiological detection are commonly used to evaluate the freshness of meat [6,7,8,9]. Due to their direct and reliable results to determine meat freshness, the three methods are widely used around the world. However, some disadvantages always exist in these methods such as errors caused by assessor fatigue, and the fact these methods are time-consuming and expensive.

In recent years, because of the development of intelligent sensory technology, electronic sensory equipment such as electronic tongues and electronic noses has shown good applicability in the detection area [10]. Based on its advantages of rapid and non-destructive detection, electronic noses have been widely used in many kinds of food evaluation, including wine discrimination [11,12,13,14,15,16], fruit quality detection [17,18,19,20,21,22,23] and meat evaluation [24,25,26,27,28,29]. Based on the previous application, we believe that the electronic nose can be used to detect the different kinds of gases produced by meat with different freshness status. In previous studies, lots of food quality features were detected by biochemical analysis methods and then used as standards for an electronic nose.

In this research, we used the human sensory method to evaluate the freshness of meat and established the freshness standard based on the sensory results. Taking this freshness standard as basis, pork, beef and mutton were taken as research samples and evaluated by the electronic nose established by our lab. The principal component analysis method and discriminant factor analysis method were used to study and analysis the freshness of the three kinds of meats.

## 2. Materials and Methods

### 2.1. Regents and Materials

**Figure 1:**
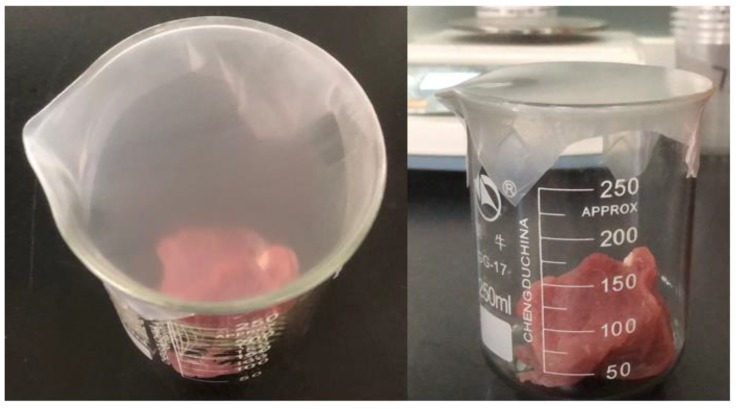
Photo of a pork sample.

The pork, beef and mutton samples were purchased from the Wumart Supermarket (Hangzhou, China). All the meats were cut into several pieces of the same weight (50 g), shape and tissue. Then, each piece of treated meat was put into a glass bottle and sealed with 3M film as shown in the Figure 1. All the treated meats were stored in a constant temperature humidity chamber (STIK Co. Ltd., Shanghai, China) at a temperature of 25 °C and 70% humidity. Different samples were used for the different days’ detection.

### 2.2. Freshness Sensory Evaluation

The sensory panel consisted of 18 experienced assessors (nine males and nine females, from 23 to 37 years old). They were explained the purpose and background of this study and were trained for two weeks (they were firstly asked to learn the color, odor, viscosity, and resilience evaluation methods and terminology; then, they were asked to observe and evaluate known samples which were provided from us; thirdly, they evaluated the unknown samples for this research). During the sensory evaluation, the assessors were asked to evaluate the color, odor and texture of meat samples after different storage times. After the evaluation, the assessors were asked to divide the samples into three groups, including fresh group, sub-fresh group and putrid group. The training and evaluation were based on the following four features:

(1) Color: muscle gloss, interstitial fluid color.
(2) Odor: meat specific odor, putrid smell.
(3) Viscosity: feel the surface viscosity and the interstitial fluid amount of the latest slice.
(4) Resilience: the recovery rate of a sunk part after pressing with the fingers.

### 2.3. Electronic Nose Detection

**Figure 2:**
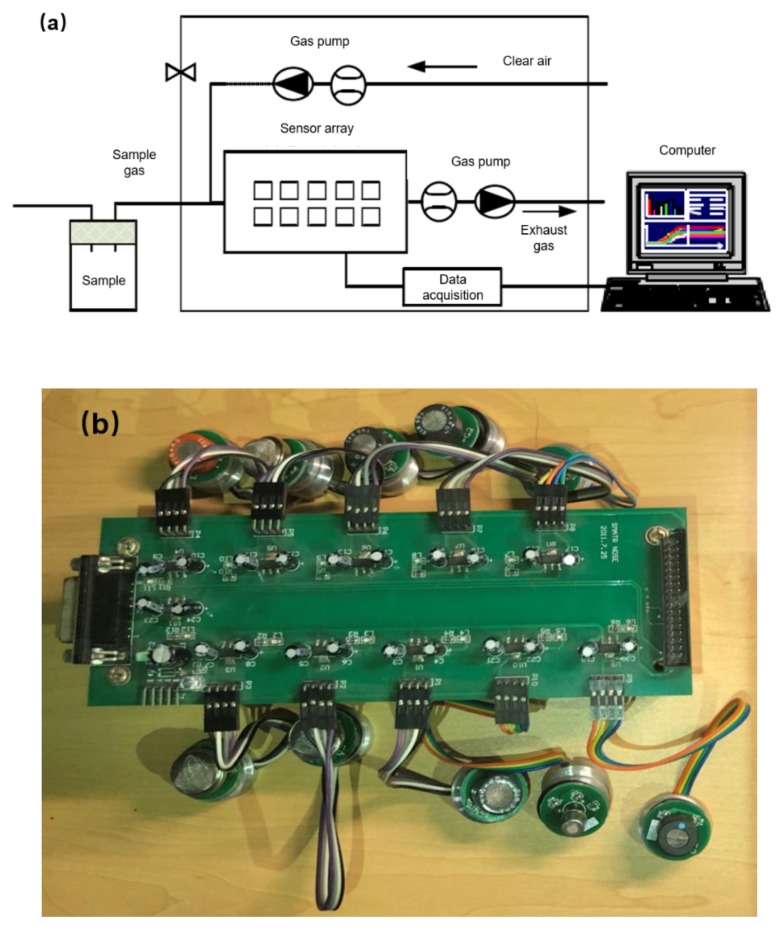
(**a**) The schematic of the electronic nose and (**b**) a photo of the sensor array.

The electronic nose (as shown in the Figure 2a) set up in our lab can be divided into five parts, including a gas injection system, gas sensor array, signal acquisition system, signal preprocessing system and intelligent pattern recognition system. The 10 sensors in the gas sensor array (as shown in the Figure 2b and listed in the Table 1), which were named from S1 to S10, were highly sensitive to amine, sulfide, organic solvent, hydrogen, hydrocarbon, inflammable gas, oxynitride, VOC and volatile gas during food cooking.

In this study, this electronic nose was used to detect samples of pork, beef and pork treated per 24 h during 7 days of storage time. The detection temperature was 40 °C. The detection and washing times were 150 s and 120 s, respectively. The washing and detection flow rates were 0.1 L/min. The electronic nose would collect the voltage value from the sensors with a samplinng rate of 100 points per second. After detection by the electronic nose, we could obtain the characteristic value (the average value of last three values of each sensor signal’s stability region) of each sample from the electronic nose which were used for further analysis.

### 2.4. Principal Component Analysis (PCA)

Pork, beef and mutton samples were detected by the electronic nose every day for six days. After detection by the electronic nose, the principal component analysis method was used to analyse the characteristic values to study the differences between samples subjected to different storage times.

### 2.5. Discriminant Factor Analysis (DFA)

Pork, beef and mutton samples with three kinds of freshness status (fresh, sub-fresh and putrid), were detected by the electronic nose. After detection, the discriminant factor analysis method was used to generate a database of the three kinds of meats with different freshness status. Then, the unknown meat samples were also detected by the electronic nose and analyzed by the discriminant factor analysis method to verify the accuracy of this database.

## 3. Results and Discussion

### 3.1. Freshness Sensory Evaluation

Eighteen experienced assessors were asked to evaluate and divide each kind of meat sample with different freshness into three groups based on the color, odor and texture. For fresh meat, the scores were equal to or greater than 70. For sub-fresh meat, the scores were lower than 70 and equal to or greater than 40. For putrid fresh, the scores were lower than 40. According to the evaluation results, which can be seen in the Table 2, we found the pork, beef and mutton samples had similar grouping situations: 1 to 2 days’ storage samples, 3 to 4 days’ storage samples and 5 to 7 days’ storage samples corressponded to the fresh group, sub-fresh group and putrid group, respectively. This sensory freshness grouping results were then used as the reference groups for the discriminant factor analysis.

### 3.2. Electronic Nose Response of Pork, Beef and Mutton Samples

**Figure 3:**
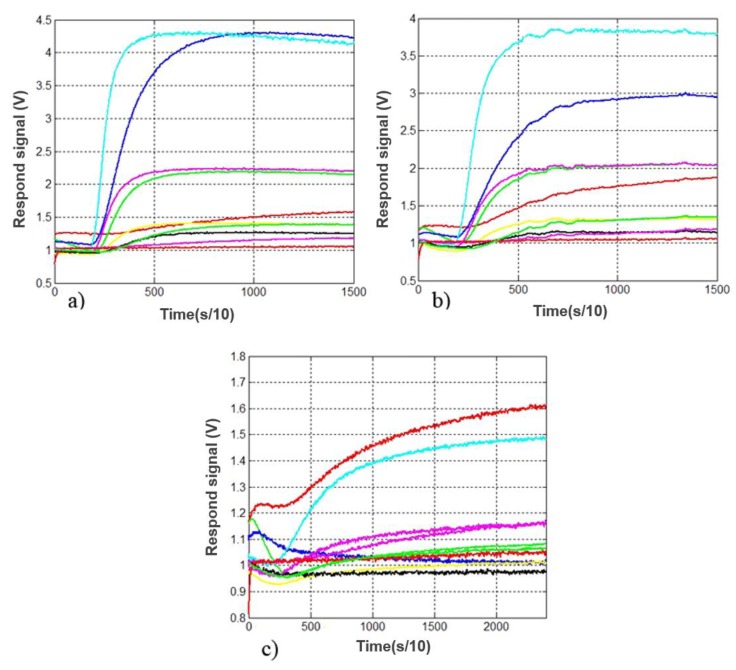
The typical original electronic nose response signals of (**a**) pork; (**b**) beef and (**c**) mutton.

The typical original electronic nose responses signal of pork, beef and mutton are shown in Figure 3, where each line in the figure represents one gas sensor. As Figure 3 shows, due to the continuously accumulation and reaction of volatile gases on the surface of the sensors, the response strength was weak at first and became stronger after 30 s. As time goes by, the response strength reached a maximum value and tends to be stable. The stabilization time was nearly 120 s for pork and beef, 220 s for mutton. The response strengths of the gas sensors which can sense hydrogen sulfide, sulfide, VOC and volatile gas during food cooking were significantly stronger than those of the other sensors. This phenomenon was consistent with the chemical reactions of stored meats due to spoilage.

### 3.3. PCA and DFA Results of Pork

**Figure 4:**
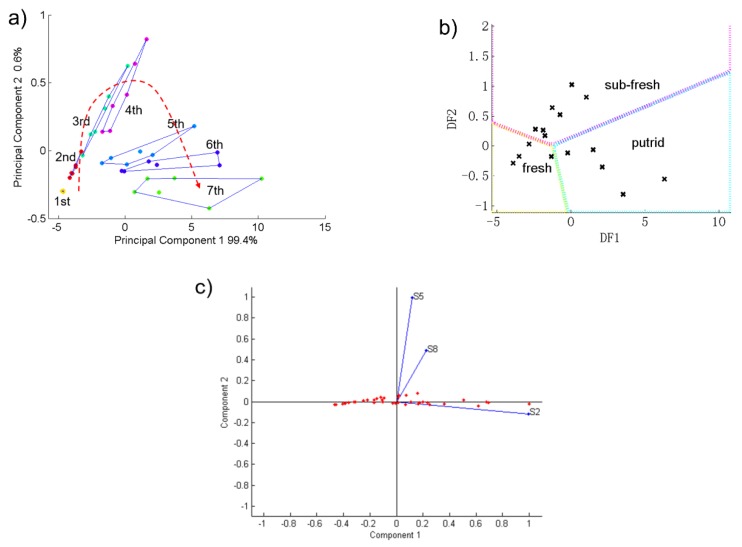
The results of pork samples with different storage time during 7 days: (**a**) PCA result; (**b**) DFA result and (**c**) Loading plot figure.

For pork, the sensor S2, S5 and S8 had stronger responses. Therefore, we used these three sensors’ data to do principal component analysis. with different freshness. Figure 4a shows the PCA figure of the pork samples’ detection result. The 1st to 7th groups in the figure represent the 1st to 7th days of storage for the pork samples. As Figure 4a shows, the contribution rates of PC1 and PC2 of the pork samples was 100%. This indicates that this electronic nose can reflect well the change trends of pork samples’ gas composition during the 7 day storage time.

Furthermore, pork samples corresponding to the same aging day in the PCA figure were independent and did not overlap with each other. On the other hand, as the storage time went by, each of the seven samples of pork in Figure 4a presents an approximately parabolic trend during the 7 day storage time. This indicates that this electronic nose could distinguish well each day’s samples of pork and display their change trend. On the other hand, Figure 4c shows that among the three most sensitive sensors, S2 was the most important sensor for distinguishing the samples.

The discriminant factor analysis method was used to generate the freshness database of pork and to classify unknown samples which were stored for different times. Based on the sensory freshness grouping result, we assigned the 7-days’ pork samples into three groups named fresh, sub-fresh and putrid using the DFA method. After establishing the database, 19 unknown pork samples with different storage times were detected by the electronic nose and distinguished by using the database. Figure 4b and Table 3 show the DFA results. Due to their respective freshness status, the 19 unknown samples were well divided into three groups as seen in Figure 4b. Table 3 lists the detailed DFA results of each unknown pork sample. Aside from the 3# and 13# samples, the other 17 unknown samples’ judgment results were correct and the total accuracy was 89.5%. Therefore, this electronic nose, with the DFA database which it was based on, can well detect, distinguish and judge pork samples’ freshness.

### 3.4. PCA and DFA Results of Beef

**Figure 5:**
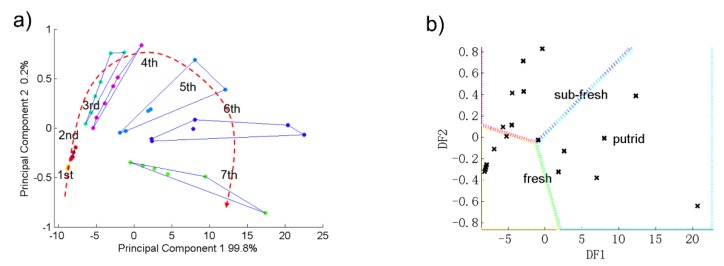

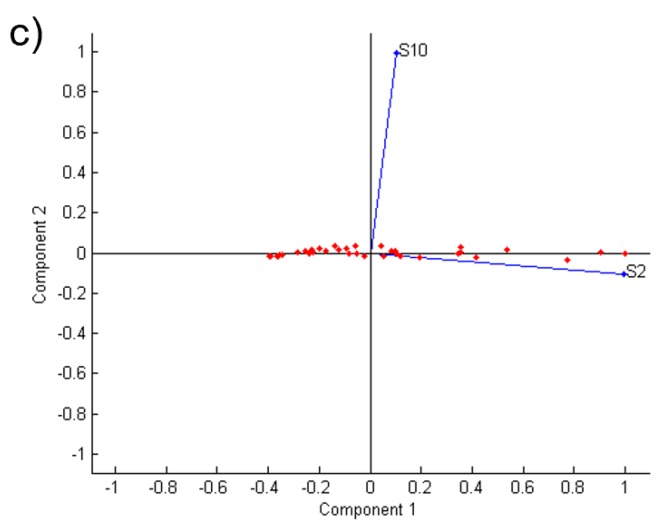
The results of beef samples with different storage times during 7 days: (**a**) PCA result; (**b**) DFA result and (**c**) Loading plot figure.

For beef, the sensors S2 and S10 had stronger responses. Therefore, we used these sensors’ data to do the principal component analysis. Figure 5a shows the PCA figure of the beef samples’ detection results. The 1st to 7th groups in the figure represent the 1st to 7th day of beef samples storage times. As Figure 5a shows, the contribution rates of PC1 and PC2 of the beef samples was 100%. This indicates that this electronic nose can reflect well the changing trends of beef samples’ gas composition during the 7 days of storage time.

Furthermore, beef samples corresponding to the same aging day in the PCA figure were independent and did not overlap each other. On the other hand, as the storage time goes by, every seven samples of beef in the Figure 5a present an approximately parabolic trend during the 7 day storage time. This indicates that this electronic nose could distinguish well each days’ beef samples and display their change trend. On the other hand, Figure 5c shows that among the two sensitive sensors, S2 was the most important sensor for distinguishing the samples.

The discriminant factor analysis method was used to generate the freshness database of beef and to distinguish the unknown samples which were stored for different times. Based on the sensory freshness grouping result, we assigned the 7-days’ beef samples into three groups, named fresh, sub-fresh and putrid using the DFA method. After establishing the database, 19 unknown beef samples with different storage times were detected by the electronic nose and classified using the database. Figure 5b and Table 4 show the DFA results 

Due to their freshness status, the 19 unknown samples were well divided into the three groups as seen in Figure 5b. Table 4 lists the detailed DFA results of each unknown beef sample. Aside from the 8#, 9# and 14# samples, the other 16 unknown samples’ judgment results were correct and the total accuracy was 84.2%. Therefore, this electronic nose, including the DFA database which it is based on, can well detect, distinguish and judge beef samples’ freshness.

### 3.5. PCA and DFA Results of Mutton

**Figure 6:**
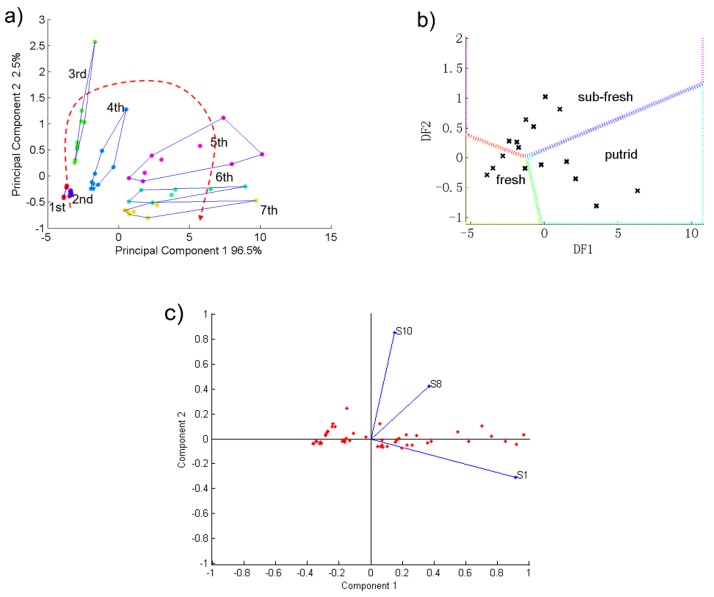
The results of mutton samples with different storage times for 7 days: (**a**) PCA result; (**b**) DFA result and (**c**) Loading plot figure.

For mutton, the sensors S1, S8 and S10 had stronger responses, therefore, we used the data from these three sensors to perform the principal component analysis. Figure 6a shows the PCA figure of the mutton samples’ detection results. The 1st to 7th groups in the figure represent the 1st to 7th day storage times of the mutton samples. As Figure 6a shows, the contribution rates of PC1 and PC2 of mutton samples was 99.0%. It indicates that this electronic nose can well reflect the changing trend of mutton samples’ gas composition during the 7 day storage time.

Furthermore, mutton samples corresponding to the same aging day in the PCA figure were independent and did not overlap each other. On the other hand, as the storage time gone on, every seven samples of mutton in Figure 6a present an approximately parabolic trend during the 7 day storage time. This indicates that the electronic nose could distinguish well each day’s samples of mutton and display their change trend. On the other hand, Figure 6c shows that among the three sensitive sensors, S1 was the most important sensor for distinguishing between the samples. The discriminant factor analysis method was used to generate the freshness database of mutton and to distinguish the unknown samples which were stored for different times. Based on the sensory freshness grouping result, we assigned the 7-day mutton samples into three groups named fresh, sub-fresh and putrid using the DFA method. After establishing the database, 19 unknown mutton samples with different storage times were detected by the electronic nose and classified using the database. Figure 6b and Table 5 show the DFA results. According to their freshness status, the 19 unknown samples were well divided into three groups as seen in Figure 6b. Table 5 lists the detailed DFA results of each unknown mutton sample. Aside from the 4# sample, the other 18 unknown samples’ judgment results were correct and the total accuracy was 94.7%. Therefore, this electronic nose, including the DFA database which it is based on, can well detect, distinguish and judge mutton samples’ freshness.

## 4. Conclusions

Pork, beef and mutton samples with different storage times were detected by an electronic nose set up in our lab. Based on the human sensory evaluation, the pork, beef and mutton samples had the same grouping situation: 1 to 2 days’ storage samples, 3 to 4 days’ storage samples and 5 to 7 days’ storage samples were defined as the fresh group, sub-fresh group and putrid group, respectively. The principal component analysis results showed that the electronic nose could well distinguish pork, beef and mutton samples with different storage times and displayed an approximately parabolic trend during 7 days of storage time. Using the sensory evaluation results as reference, the discriminant factor analysis showed the electronic nose could well distinguish and judge the freshness of samples (the accuracy was 89.5%, 84.2% and 94.7% for pork, beef and mutton, respectively), indication this electronic nose is promising for meat fresh detection applications.

## Figures and Tables

**Table 1:** Gas sensor array and its properties.

Sensor Number	Sensitive Substances
S1	methane, biogas
S2	alcohols, ketones, aldehydes, aromatic compounds
S3	hydrogen sulfide, sulphide
S4	ammonia gas, amines
S5	hydrogen
S6	alcohol, organic solvent
S7	combustible gas
S8	VOC
S9	cooking odors
S10	alcohol, organic solvent

**Table 2:** Human sensory evaluation results of pork, beef and mutton.

Meat	Sensory Evaluation Score						
	Day 1	Day 2	Day 3	Day 4	Day 5	Day 6	Day 7
Pork	85	74	63	48	39	32	22
Beef	89	76	66	45	38	34	25
Mutton	82	71	60	42	37	29	21
Freshness	Fresh		Sub-fresh		Putrid		

**Table 3:** DFA result of 19 unknown pork samples.

Sample Number	Storage Time	DFA Result	Accuracy	Total Accuracy
1	2 days	Fresh	Correct	89.5%
2	2 days	Fresh	Correct	
3	2 days	Sub-fresh	Wrong	
4	2 days	Fresh	Correct	
5	3 days	Sub-fresh	Correct	
6	3 days	Sub-fresh	Correct	
7	3 days	Sub-fresh	Correct	
8	4 days	Sub-fresh	Correct	
9	4 days	Sub-fresh	Correct	
10	4 days	Sub-fresh	Correct	
11	5 days	Putrid	Correct	
12	5 days	Putrid	Correct	
13	5 days	Fresh	Wrong	
14	6 days	Putrid	Correct	
15	6 days	Putrid	Correct	
16	6 days	Putrid	Correct	
17	7 days	Putrid	Correct	
18	7 days	Putrid	Correct	
19	7 days	Putrid	Correct	

**Table 4:** DFA results of 19 unknown beef samples.

Sample Number	Storage Time	DFA Result	Accuracy	Total Accuracy
1	2 days	Fresh	Correct	84.2%
2	2 days	Fresh	Correct	
3	2 days	Fresh	Correct	
4	2 days	Fresh	Correct	
5	3 days	Sub-fresh	Correct	
6	3 days	Sub-fresh	Correct	
7	3 days	Sub-fresh	Correct	
8	3 days	Fresh	Wrong	
9	4 days	Fresh	Wrong	
10	4 days	Sub-fresh	Correct	
11	4 days	Sub-fresh	Correct	
12	4 days	Sub-fresh	Correct	
13	5 days	Putrid	Correct	
14	5 days	Sub-fresh	Wrong	
15	6 days	Putrid	Correct	
16	6 days	Putrid	Correct	
17	7 days	Putrid	Correct	
18	7 days	Putrid	Correct	
19	7 days	Putrid	Correct	

**Table 5:** DFA results of the 19 unknown mutton samples.

Sample Number	Storage Time	DFA Result	Accuracy	Total Accuracy
1	1 days	Fresh	Correct	94.7%
2	1 days	Fresh	Correct	
3	1 days	Fresh	Correct	
4	2 days	Fresh	Correct	
5	2 days	Fresh	Correct	
6	3 days	Sub-fresh	Correct	
7	3 days	Sub-fresh	Correct	
8	3 days	Sub-fresh	Correct	
9	4 days	Fresh	Wrong	
10	4 days	Sub-fresh	Correct	
11	4 days	Sub-fresh	Correct	
12	5 days	Putrid	Correct	
13	5 days	Putrid	Correct	
14	6 days	Putrid	Correct	
15	6 days	Putrid	Correct	
16	6 days	Putrid	Correct	
17	7 days	Putrid	Correct	
18	7 days	Putrid	Correct	
19	7 days	Putrid	Correct	
